# Structural Damage Localization and Quantification Based on a CEEMDAN Hilbert Transform Neural Network Approach: A Model Steel Truss Bridge Case Study

**DOI:** 10.3390/s20051271

**Published:** 2020-02-26

**Authors:** Asma Alsadat Mousavi, Chunwei Zhang, Sami F. Masri, Gholamreza Gholipour

**Affiliations:** 1 School of Civil Engineering, Qingdao University of Technology, Qingdao 266033, China; 2 Department of Civil Engineering, University of Southern California, Los Angeles, CA 90089-2531, USA

**Keywords:** damage detection, signal processing, steel-truss bridge, artificial neural network, Hilbert–Huang Transform (HHT), complete ensemble empirical mode decomposition with adaptive noise (CEEMDAN)

## Abstract

Vibrations of complex structures such as bridges mostly present nonlinear and non-stationary behaviors. Recently, one of the most common techniques to analyze the nonlinear and non-stationary structural response is Hilbert–Huang Transform (HHT). This paper aims to evaluate the performance of HHT based on complete ensemble empirical mode decomposition with adaptive noise (CEEMDAN) technique using an Artificial Neural Network (ANN) as a proposed damage detection methodology. The performance of the proposed method is investigated for damage detection of a scaled steel-truss bridge model which was experimentally established as the case study subjected to white noise excitations. To this end, four key features of the intrinsic mode function (IMF), including energy, instantaneous amplitude (IA), unwrapped phase, and instantaneous frequency (IF), are extracted to assess the presence, severity, and location of the damage. By analyzing the experimental results through different damage indices defined based on the extracted features, the capabilities of the CEEMDAN-HT-ANN model in detecting, addressing the location and classifying the severity of damage are efficiently concluded. In addition, the energy-based damage index demonstrates a more effective approach in detecting the damage compared to those based on IA and unwrapped phase parameters.

## 1. Introduction

### 1.1. Background

Damage detection and operational safety assessments of bridges as the strategic civil infrastructures have always been vital challenges in structural health monitoring (SHM). Nowadays, the time-frequency based methods of signal processing as a part of vibration characteristic-based techniques are increasingly utilized by researchers. Among the various parts of existing approaches, signal processing techniques are known as the key components that are frequently used in scientific and engineering fields. The signal processing techniques in SHM are firstly based on extracting the fundamental parameters of acquired structural vibration responses under various demands (e.g., loading scenarios, damage or health status, etc.). Then, the characteristics of these parameters are interpreted to monitor the behavior of a structure for the sake of preserving the structural performance during its service life.

Generally, conventional signal processing techniques such as Fast Fourier Transform (FFT) [1,2,3], and Short-Time Fourier Transform (STFT) [4,5] are employed to analyze the linear and stationary signals. However, the responses of structures in the real world are inherently nonlinear according to structural health monitoring systems [6,7] and non-stationary. Therefore, the need for adopting Hilbert–Huang Transform (HHT) [8,9] has been proposed which is able to perfectly evaluate the nonlinear and non-stationary vibrations. The procedure of this technique contains two steps: (i) to decompose the original signal by the empirical mode decomposition (EMD) method into a series of complete and oscillatory components, named intrinsic mode functions (IMFs), and (ii) to capture the instantaneous frequency and amplitude features by applying Hilbert transform (HT) to the IMFs. The vast majority of studies in the literature utilized HHT based on EMD for SHM and damage detection purposes [10,11,12,13,14,15,16].

Pines and Salvino [11] proposed a signal processing method based on the analysis of time-series data from a one-dimensional scaled civil building which was tested for the cases with and without structural damage. From the results, it was found that this method was able to address the unique features of the vibratory response of the structure. Carbajo et al. [15] proposed a new automated damage identification strategy based on the extracted sensitive features of windowed HHT including the instantaneous frequency and energy, from an experimental single degree of freedom (DOF) system and a wind turbine blade. Quek et al. [17] demonstrated the viability of HHT for locating an anomaly in the form of different structural members such as sandwiched aluminum beams, reinforced concrete (RC) slabs, and plates, with regards to multifarious damage levels. Liu et al. [18] concluded the efficiency of the HHT method in detecting and locating the damage in a scaled four-story structure through the comparison of extracted frequencies from the first three IMFs for healthy and damaged states. Yang et al. [19] employed the EMD and Hilbert transform to determine the damage time instant, the natural frequencies, damping ratios, and damage locations, before and after damage statuses of an American Society of Civil Engineers (ASCE) benchmark structure. The damage scenarios were accomplished by applying sudden changes in the stiffness of the structure. In their study, the presence of damage was recognized by observing the damage spikes in the signals using EMD.

Although EMD has been widely used in various fields of engineering [20,21,22,23,24,25] due to its unique properties and preferable performance, it still suffers from the lack of firm theoretical basis and analytical interpretation of the outputs. Hence, it is extremely needed to recognize the statistical characteristics of this technique in terms of both model selection and parameter estimation for different applications in the real world. There exist many research works in the literature focusing on various statistical techniques [26,27,28,29] to obtain IMFs from the EMD. The statistical characteristics of IMFs of white Gaussian noise were studies by Wu and Huang [30] using a Gaussian distribution. In their proposed method, the mean of energy and periods of IMFs were considered to investigate the properties of IMFs. In an unlikely development, the privilege of Laplacian distribution above a Gaussian distribution for IMFs was revealed by Schlotthauer et al. [31]. In addition, the significant influences of the sample size and the number of sifting iterations on the probability of density functions (PDFs) of IMFs were concluded. As another statistical technique in obtaining the EMD, a two-step entropic method was utilized by Tseng and Lee [32] to analyze the characteristics of IMFs using a Bayesian interpretation. In the first step, the resulting IMFs were clustered into two groups, and then, the relative entropy was applied between the input and clustered data during the second step. A nonlinear Bayesian filtering framework was also adopted by Sameni et al. [33] in the denoising process of signals. The superiority of this filtering approach compared to conventional filtering methods was concluded.

Despite many applications of EMD in the literature, this method suffers some drawbacks such as generating undesirable IMFs at the low-frequency region with the mode mixing problem leading to inaccurate SHM results. To overcome this, Torres et al. [34] proposed the complete ensemble empirical mode decomposition with adaptive noise (CEEMDAN) by adding a particular noise in which, after each stage of the decomposition process, a unique residue is obtained.

In addition, Artificial Neural Network (ANN) has been widely used in SHM to automate the damage identification approach with the purpose of effectively reducing the human intervention and speeding up the process of structure diagnosis [35]. Most of the previous research works adopted the structural response parameters to train the ANN for the identification of the damages in civil engineering structures [36,37,38,39]. An ANN-based structural damage detection approach was presented by Masri et al. [40] to monitor the linear and nonlinear systems through structural parameters even in the presence of noisy environments. Thereafter, Masri et al. [41] proposed a nonparametric damage detection approach based on nonlinear identification methods for the structural unknown system through the ANN model. Dackermann et al. [42] utilized cepstrum analysis and artificial neural networks to determine the structural parameter relying on response-only measurements and damage detection procedures, respectively. Qian and Mita [39] proposed an acceleration-based damage evaluation methodology for a building structure subjected to various excitation and damage levels using ANN to detect the location and severity of the damage. The performance of ANN was assessed by the Relapsing-Remitting Multiple Sclerosis (RRMS) damage indices, and its effectiveness was concluded. Arangio and Bontempi [43] utilized Bayesian ANN based on mode shape and frequency features for long-term monitoring and damage identification of a cable-stayed bridge as a real benchmark case study. The capability of the proposed method in detecting the damage was found compared to the traditional vibration-based techniques. Zang et al. [44] assessed the advantages of combining independent component analysis (ICA) and ANN in the damage detection procedure of two example models of truss and three-story bookshelf structure. Experimental results demonstrated the effectiveness of the proposed methodology.

One of the significant issues in vibration-based structural damage detection is to construct and extract the sensitive parameters from structural dynamic responses to identify the initial damages. To this end, a multifarious structural damage identification system based on the combination of signal processing and artificial intelligence techniques has been developed recently. This can guarantee the robustness, reliability, and efficiency of the detection process [45]. Garcia-Perez et al. [46] combined wavelet packet transform (WPT) and EMD techniques and utilized ANN to locate and detect the combined damage of a five-bay truss-type structure. Xun and Yan [47] evaluated the capability of the combination of radial basis function neural network (RBFNN) and HHT in vibration signal analysis. RBFNN was used as a pre-processor to extend the length of the signal for removing the end swings problem and as a post-processor to select the optimal IMFs. Vazirzade et al. [48] studied the reliability of modal analysis using HHT and ANN to detect the damage of a three-story moment-resisting frame. In their methodology, the first-mode frequency and mode shape were captured through the ensemble empirical mode decomposition (EEMD) and HT methods; then, ANN determined the location and severity of the damage.

Conventional classification techniques such as Autoregressive Integration Moving Average (ARIMA) and Exponential smoothing (ES) are fitted to time series data either to better understand the data or to predict future points in the series (forecasting). Basically, the forecasting process depends on the length of the series and properties. If time-series data set are short and have a trend, then ARIMA or ES can be used as a classical method. These classical techniques are suitable to analyze the short data set and take care of trends, seasonality, and cycles. These techniques are commonly used for the forecast in finance and economics. For long time-series with seasonality, Artificial Neural Network is used as a powerful machine learning method. ANN has been a better choice for researchers in structural and tools condition monitoring because of its advantages such as superior learning, noise suppression, and parallel computation. One of the limitations associated with the ARIMA model is that it is not able to capture the nonlinear pattern of the time series variable reflected in the nonlinear and non-stationary responses of structures in the real world [49]. In addition, the ES method is the only class of linear model that can only capture the linear feature of time series. Since the time series of the responses of real-world structures are often full of nonlinearity and irregularity, the ES model is unable to find accurate nonlinear patterns in the time series data [50]. In SHM and damage diagnosis of complex structures such as bridges in which data sets are long and big, ANNs are flexible and suitable machine learning to speed-up the damage diagnosis process. In addition, they enhance the ability of damage detection procedure to learn by example, which make them very flexible and powerful. Through a comprehensive study on machine learning techniques existing in the literature, ANN is more compatible with the extracted data set of the truss.

A multilayer feed-forward neural network is an interconnection of perceptron in which data and calculations flow in a single direction, from the input data to the outputs. The number of layers in a neural network is the number of layers of the perceptron. Fundamentally, feed-forward models of ANN are the first and simplest type of ANN, and are commonly used for damage identification of civil structures that take fixed sizes for input and output data. Although a recurrent neural network (RNN) [51] is a type of ANN, it is applicable in cases in which the input/output size is variant. In this study, since the length of the input/output data set is fixed on 2000 data points, ANN is therefore adopted as a supervised machine learning with the purpose of automatic damage identification, optimization, control, and prediction. The use of ANN can effectively speed up the process of structural damage diagnosis and reduce the human factor. Employment of such neural network techniques allows for predicting the structural health during the service without the need to interrupt or terminate the usage of the structure.

### 1.2. Motivation

Since the use of the HHT method combined with neural networks is very limited in the literature, this paper aims to evaluate the performance of a proposed methodology from the combination of CEEMDAN-Hilbert transform and neural network techniques called CEEMDAN-HT-ANN, which has not been reported for damage detection in civil engineering. Due to the potential advantages of the CEEMDAN demonstrated by Torres et al. [34] compared to the previous generation of EMD-based techniques, CEEMDAN is employed to decompose the acceleration response of a steel-truss bridge model which was experimentally established in laboratory settings, and subjected to a band-limited white noise excitation. The vast majority of previous researchers used modal parameters for structural health monitoring. In addition, evaluating the signal features in both time and frequency domains all at once is a gap of knowledge in SHM and damage identification in civil engineering applications, especially for complex structures such as bridges. The change of structure stiffness that would occur due to structural damage would substantially cause the potential changes and unexpected anomalies in these features. Therefore, it aims to qualitatively and quantitatively assess the sensitivity of these features to the presence, location, and severity of the damage in the truss as the evaluation criteria for damage detection in this paper. The main advantage of this study is the use of four signal features including energy, instantaneous amplitude (IA), unwrapped phase, and instantaneous frequency (IF) that extracted through applying the Hilbert transform on decomposed IMFs by CEEMDAN. These features have not been evaluated all at once in damage detection procedure in civil engineering applications. Indeed, from an extensive review on the evaluation of these features, it was concluded that the change of the structure stiffness (i.e., the occurrence of damage) extremely affects the output results based on the aforementioned features. Therefore, these signal features are utilized in damage evaluation and identification through the proposed methodology. In addition, adopting ANN as a supervised machine learning with the purpose of automatic signal analysis and damage identification can effectively speed up the process of structure diagnosis and reducing the human factor.

The uniqueness and special advantages of using the HHT based on CEEMDAN compared to conventional techniques such as FFT or wavelet is the feasibility of analyzing the nonlinear and nonstationary data and extracting the key features of the decomposed signal including IA, energy, unwrapped phase, and IF. These characteristics of IMFs can reflect how the energy and phase of the signal vary with time. Moreover, the IF feature indicates the data in a time-frequency-power domain through the spectrogram plots. In addition, since the IMF is almost mono-component, all the instantaneous parameters from a nonlinear and non-stationary signal can be efficiently captured by HT. The information obtained by these features can provide a more accurate and real-life representation of the signal which can overcome some shortcomings such as artifacts associated with the nonlocal and adaptive limitations generated by FFT and wavelet methodologies.

Section 2 of this paper provides a brief overview of the relevant mathematical background of the specific signal processing approaches adopted in the present work to analyze the experimental measurements. Section 3 describes the experimental setup of the truss steel bridge as a case study and the application methodology of CEEMDAN in the damage detection process. Section 4 provides discussions on the test results and the assessments of the sensitivity of different approaches in the damage detection and quantification. Finally, the summary and conclusions are presented in Section 5.

## 2. Theoretical Background

### 2.1. Complete Ensemble Empirical Mode Decomposition with Adaptive Noise

Owing to some major drawbacks such as mode mixing problem existing in EMD, residual noise in IMF, and difficulty in averaging of a different number of IMFs caused by adding different white Gaussian noise to the signal in EEMD technique, Torres et al. [34] solved these faults by proposing the CEEMDAN. CEEMDAN has a different trend from EEMD in adding a particular noise *E_j_*[*w_i_*(*t*)] at each step of the decomposition process, instead of adding the white Gaussian noise that is obtained with a unique residue after the extraction of each IMF. The CEEMDAN method can be defined as the following steps:

1. Add E1(wi(t)) (i = 1, 2, …, I), to the initial signal, x(t), where wi, β, and I indicate the ith added white Gaussian noise, the amplitude of the ith added white noise, and ensemble size, respectively:
  *x_i_(t)* = *x(t)* + *β_0_E_1_*(*w_i_(t)*) (1)
2. Calculate the first *IMF* (${\bar{c}}_{1}\left(t\right)$) through the first residue (i.e., *r_1_*(*t*)) as follows:
  (2) $${\bar{c}}_{1}(t)=x(t)-r_{1}(t),\;\mathrm{where}\;r_{1}(t)=\frac{1}{I}\sum_{i=1}^{I}M(x_{i}(t))$$
  where *M* (·) is the operator that represents the local means of a signal.
3. Obtain the second IMF ${\bar{c}}_{2}(t)=r_{1}(t)-r_{2}(t)$, where $r_{2}(t)=\frac{1}{I}\sum_{i=1}^{I}M\left(r_{1}(t)+\beta_{1}E_{2}(w_{i}(t))\right)$, and *E_2_*(*w_i_*(*t*)) is the second IMF of EEMD.
4. Repeat Step 3 to obtain *j*th IMF of CEEMDAN ${\bar{c}}_{j}(t)=r_{j-1}(t)-r_{j}(t)$ where
  (3) $$r_{j}(t)=\frac{1}{I}\sum_{i=1}^{I}M\left(r_{j-1}(t)+\beta_{j-1}E_{j}(w_{i}(t))\right)$$
  where *β_j_ = ε_0_*std[*r_j_*(*t*)] is the signal-noise ratio (SNR).

Since the CEEMDAN is an empirical method, it needs a trial and error procedure to obtain a good decomposition of the signal. For this reason, the CEEMDAN method converges the results based on noise standard deviation (Nstd), the number of realizations (NR), and the maximum number of sifting iterations (MaxIter). In this study, the convergence values for these parameters are Nstd = 0.2, NR = 100, and MaxIter = 1000.

### 2.2. Hilbert–Huang Transform (HHT)

The Hilbert–Huang transform (HHT) that was developed by Huang et al. [8] as an adaptive and capable tool for the non-stationary and nonlinear situations includes two procedures. In the first procedure, the empirical mode decomposition (EMD) decomposes a signal into a finite set of intrinsic components called “intrinsic mode function” (IMF). Then, the Hilbert Transform (HT) is applied to each IMF to obtain instantaneous frequencies (IF) and instantaneous amplitude (IA) features. This eventually yields a time-frequency representation (Hilbert spectrum) for each IMF. According to [8], for a signal *X*(*t*), HT is obtained, Y(t), as:

(4) $$Y(t)=\frac{1}{\pi }P\int_{-\infty }^{\infty }\frac{X(t^{\prime })}{t-t^{\prime }}\;dt^{\prime }$$

where *P* is the Cauchy principal value, and *t* is the time variable and *t*’ is the time interval.

Subsequently, the HHT of the signal is given as follows:

(5) $$Z(t)=X(t)+iY(t)=a(t)\;e^{i\theta (t)}$$

where a(t) and θ(t) are the amplitude and phase, respectively, which are defined as:

(6) $$a(t)=\sqrt{X^{2}(t)+Y^{2}(t)}\;,\;\theta (t)=\arctan\left(\frac{Y(t)}{X(t)}\right)$$

In addition, the instantaneous frequency (IF) is the derivative of the phase function:

(7) $$f(t)=\frac{1}{2\pi }\frac{d\theta }{dt}$$

Since Equation (7) is restricted to interval lengths of 2π, it is not able to capture the continuity of the phase. Hence, the unwrapped phase function is used to preserve this continuity, which obtains a monotonic increase.

### 2.3. Applications of ANNs

The basic idea of using ANN as a supervised machine learning tool in this paper is to establish an artificial intelligence component in identifying the structural damage through its pattern recognition. The training procedure of the learning machine is on the basis of the iterative calculation of parameters identified in the network to reach the converged outputs by minimizing the compute error between the target and obtained network outputs. The training test in the network is carried out based on the prediction process for the new input data set, which is called “network generalization”. To this end, a feed-forward multilayered perceptron (FFMLP) architecture is used to predict the presence of damage in the truss bridge. The multi-layer perceptron (MLP) architecture includes an input layer, one or more hidden layers, and an output layer, each of which contains a set of various nodes that learned using the Levenberg–Marquardt algorithm. In this paper, feed-forward networks include twenty hidden layers in which the input data are trained through the backpropagation algorithm. To do this, the Neural Networks Toolbox of MATLAB is used to implement the ANN simulation. In order to activate the neurons in the hidden and input layers, a log-sigmoid transfer function written as *f* (*N*) = 1/[1+exp(−*N*)] is used in which *N* is the number of neurons in layer. In addition, the *trainlm* function is used according to the resilient backpropagation algorithm for training of the networks. The accuracy of the neural network utilized in this study is examined by the Mean Square Error (MSE) method as shown in Figure 7.

Before using the FFMLP ANN to diagnose the health condition of the bridge, first, the acceleration responses of the truss before and after damage scenarios are decomposed through CEEMDAN to generate IMFs. Then, the Hilbert transform is applied to each IMF to extract key parameters including instantaneous amplitude (IA), energy, unwrapped phase, and IF. The obtained IMFs and the four aforementioned features are respectively used as the input and target layers to train the ANN in a healthy state as a reference condition of the truss. That is, the training process of the ANN is based on the healthy state of the truss bridge. The sizes of the input, hidden, and output nodes in the proposed ANN are 10, 20, and 4, respectively. In implementing this methodology, IMFs 1 to 10 decomposed by CEEMDAN for each of 13 sensors are selected as the inputs to the network and four extracted parameters including energy, IA, unwrapped phase, and IF are selected as the outputs of the network. Finally, the extracted IMFs from the acceleration responses of damaged truss through various damage scenarios are used to test the trained ANN.

### 2.4. Damage Indices Based on the CEEMDAN-HT-ANN Model

After training and testing the CEEMDAN-HT-ANN model as described in detail in Section 2.3, the differences between the outputs of the model for healthy and damaged states of the truss indicate the presence of the damage based on IA, energy, unwrapped phase, and IF parameters. In this paper, three different damage indices (DIs) based on energy, IA, and unwrapped phase are defined to detect, locate, and classify the severity of damage in a steel-truss bridge to compare the differences between the outputs of the CEEMDAN-HT-ANN model for the healthy and damaged states. It should be noted that the high scalar values of DIs represent the existence of the damage. Similarly, for locating the damage on the truss, the higher values of DIs for the sensors that are close to the location of the damaged element indicate the location of the damage. In addition, it is expected to increase the damage index values by increasing the damage severity.

The damage index based on the energy parameter has been widely used in the literature [52,53]. The energy (*E*) of signals is defined as the sum of the square of the IMFs given as follows:

(8) $$E=\int_{0}^{t_{0}}{\left(IMF\right)}^{2}dt$$

where the parameter *E* is selected as one of the parameters in the output layer.

The IMFs and the energy are used as the input and target layers for the healthy state of the truss in the training process of the ANN, respectively. Thereafter, the IMFs extracted from the acceleration responses of the damage scenarios are used to test the ANN model. Then, the energy-based damage index compares the differences between the outputs of the CEEMDAN-HT-ANN model for the healthy and damaged states defined as follows:

(9) $$DI_{\left(E\right)}=\left|\frac{E_{Healthy}-E_{Damaged}}{E_{Healthy}}\right|\times 100$$

*E_Healthy_* and *E_Damaged_* represent the output of the CEEMDAN-HT-ANN model based on the acceleration response of the healthy and damaged states, respectively.

The similar procedure as defined for the energy feature is employed on the IA parameter extracted through applying of Hilbert transform (HT) to each IMF and calculating the average of the IMFs given as follows:

(10) $$\bar{I}{\bar{A}}_{IMF}=\frac{1}{n}\sum_{i=1}^{n}\bar{I}{\bar{A}}_{i}$$

where $\bar{I}{\bar{A}}_{i}=\left[\bar{I}{\bar{A}}_{1},\bar{I}{\bar{A}}_{2},\ldots ,\bar{I}{\bar{A}}_{n}\right]$ represents the instantaneous amplitude of each IMF extracted by HT, and *n* is the number of time samples. The IMFs and *IA_IMF_* are used as the input and target layers of a healthy state of the truss in the training process of the ANN, respectively. Then, the IMFs of the damage scenarios are used to test the ANN. Accordingly, the damage index based on IA can be defined as follows:

(11) $$DI_{\left(IA\right)}=\left|\frac{\bar{I}{\bar{A}}_{Healthy}-\bar{I}{\bar{A}}_{Damaged}}{\bar{I}{\bar{A}}_{Healthy}}\right|\times 100$$

*IA_Healthy_* and *IA_Damaged_* represent the output of the CEEMDAN-HT-ANN model based on the acceleration response of the healthy and damaged states, respectively.

As discussed in Section 2.2, *θ*(*t*) is the phase of the IMFs calculated by Equation (6). The same procedure is carried out for the unwrapped phase parameter (*P*) extracted by applying the Hilbert transform (HT) to each IMF and calculating the average of the IMFs given as follows:

(12) $${\bar{P}}_{IMF}=\frac{1}{n}\sum_{i=1}^{n}P_{i}$$

where $P_{i}=\left[P_{1},P_{2},\ldots ,P_{n}\right]$ denotes the unwrapped phase of each IMF extracted by HT.

The IMFs and *P_IMF_* are used as input and target layers of the healthy state of the truss in the training process of the ANN, respectively, then the IMFs of the damage scenarios are used to test the ANN. Accordingly, the damage index based on *P* can be defined as follows:

(13) $$DI_{\left(P\right)}=\left|\frac{P_{Healthy}-P_{Damaged}}{P_{Healthy}}\right|\times 100$$

*P_Healthy_* and *P_Damaged_* represent the output of the CEEMDAN-HT-ANN model based on the acceleration response of the healthy and damaged states, respectively.

## 3. Applications

### 3.1. Experimental Setup

**Figure 1:**
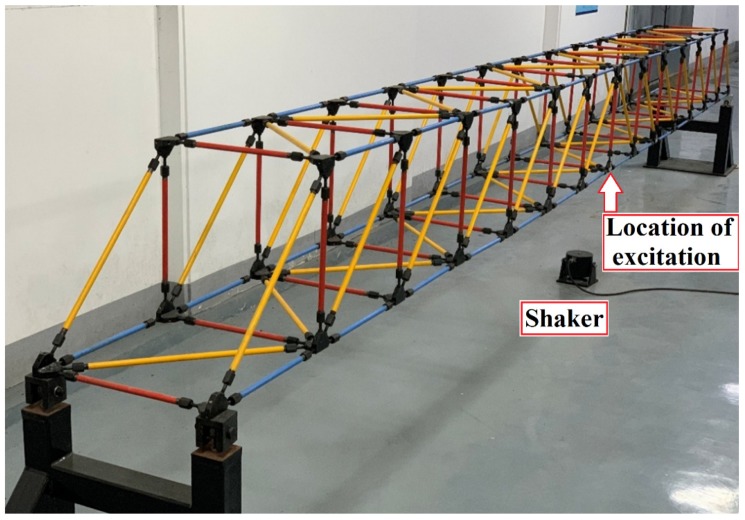
A perspective view of the steel truss bridge model as the case study in the present work established in the Qingdao University of Technology (QUT) laboratory.

**Figure 2:**
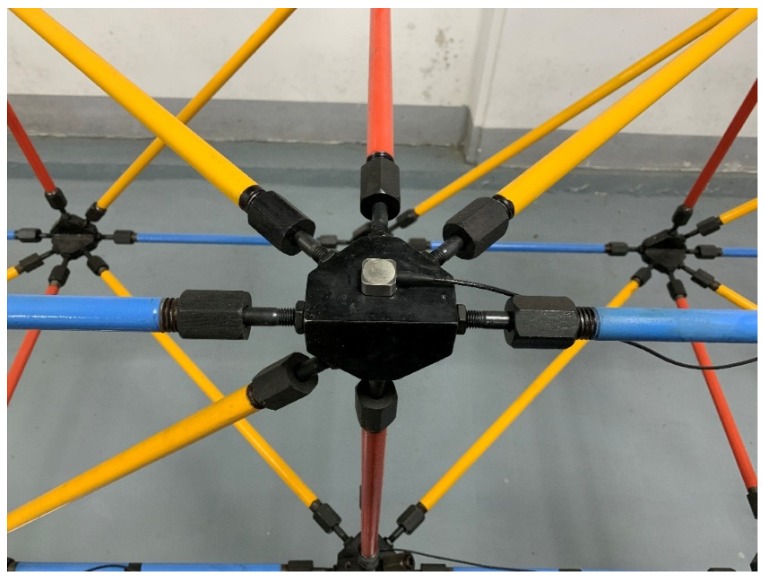
Details of a typical joint with the attached acceleration sensor.

A fourteen-bay steel truss bridge with a span length of 5.6 m, as shown in Figure 1, which was experimentally established in the Qingdao University of Technology (QUT), is considered as the case study in this paper. This model was built according to the design specifications presented by the Smart Structures Technology Laboratory (SSTL) of the University of Illinois at Urbana-Champaign [54]. The length of all the horizontal and vertical members is 0.4 m on each side while the length of all diagonal members is 0.4√2 m. The truss is pin-supported in one end, and roller-supported in the other end. All members are steel tubes with an inner diameter of 1.09 cm and an outer diameter of 1.71 cm. truss elements are connected to each other by screwing the collars at the joint, as shown in detail in Figure 2. This connection allows for easily replacing the elements to simulate the damage scenarios at the desired locations.

**Figure 3:**
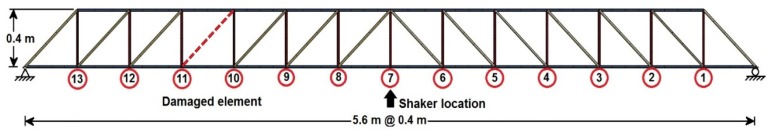
A schematic view of the truss bridge model by showing the uniform distribution of the locations of sensors, and the location of the damaged element.

The excitation load is vertically applied at the bridge mid-span using a shaker (CF6900-100), as shown in Figure 3, which can generate a maximum force of 100 N, and vibrations with frequency ranges between 10 and 2500 Hz. A band-limited white noise is generated using MATLAB software with an RMS value of 0.4 and a bandwidth of 1.0 Hz. Then, an Arbitrary Function Generator (AFG1022) is used to drive the shaker. AFG1022 is applied to the bridge through a steel rod connected to the shaker. The amount of transferred force is measured using an attached load cell (CF3110) on the head of this rod, with a sensitivity of 25.32 mV/N. Additionally, a power amplifier device (CF6502) is used to magnify and control the input voltage from AFG to the shaker.

In addition, the response of the structure is measured using a series of piezotronic accelerometers (herein, 13 pieces) (CF0420), with a sensitivity of 60 mV/g and frequency ranges from 0 to 1400 Hz. The accelerometers are placed at the joints of the lower chord of the bridge with a uniform distribution as illustrated in Figure 3. The recorded data by each of the accelerometers are separately inputted to each of eight channels of a series of synchronized data acquisition devices (CF3820) for measuring. The data are acquired with a sampling accuracy of 2000 points per second by the data acquisition software.

**Figure 4:**
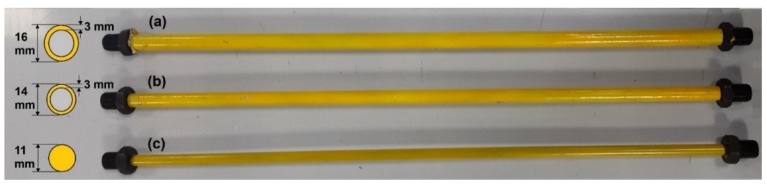
Details of the damaged elements with; (**a**) 20%, (**b**) 50%, and (**c**) 80% reduced percentages of the cross-section stiffness.

In this study, the damaged states of the truss are simulated by reducing the cross-sectional stiffness or removing a diagonal element as shown in Figure 3. It is worth being noted that the severity of damage is handled through the cross-section reduction scenarios. To do this, three different damage levels are considered by reducing the cross-section stiffness (i.e., the moment of inertia). The original member identified in Figure 3 is replaced by three damaged elements with 20%, 50%, and 80% reduced percentages of cross-section stiffness, as shown in Figure 4. All other structural parameters and loading conditions are kept constant for all scenarios studied in this paper.

### 3.2. Procedure of the CEEMDAN-HHT-ANN Damage Detection Method

**Figure 5:**
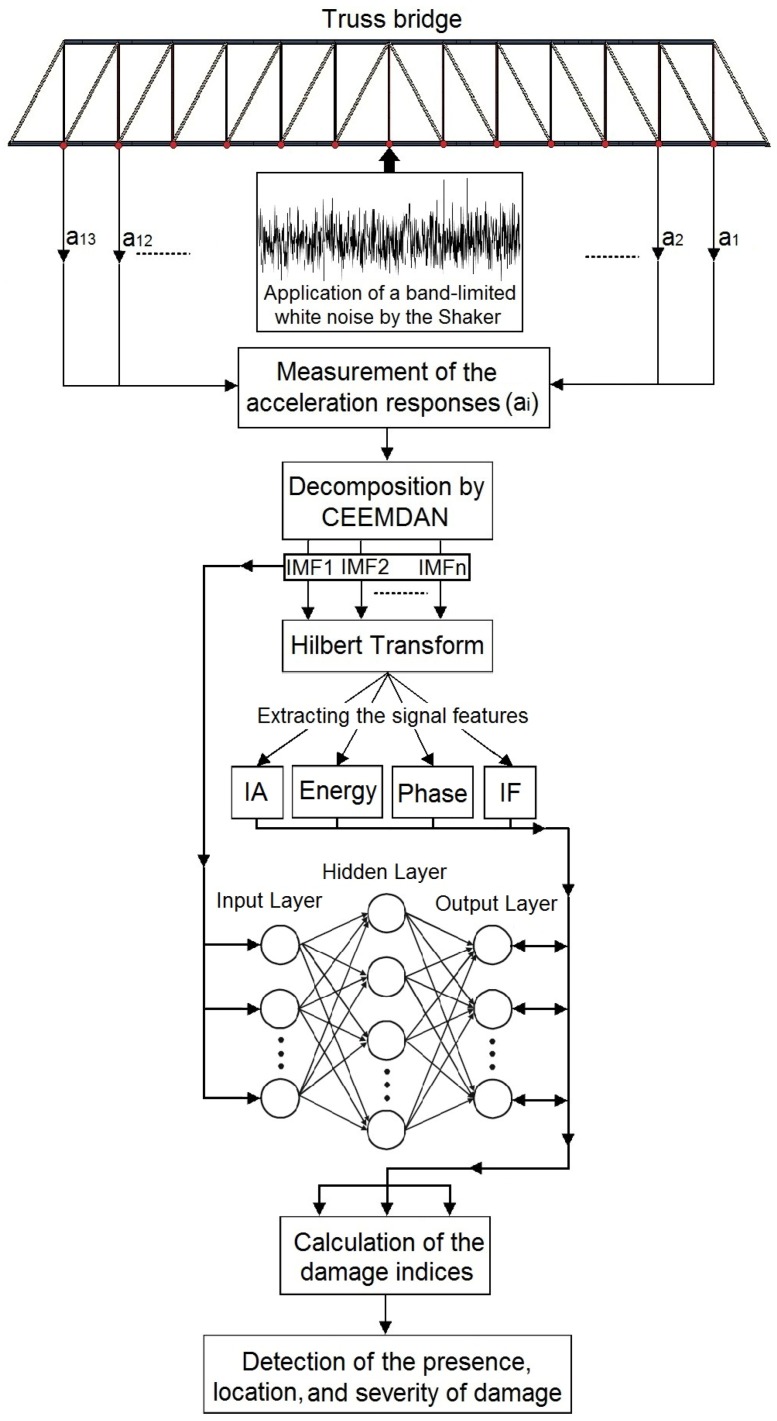
The framework flowchart of the proposed CEEMDAN-HT-ANN model to detect, locate, and classify the severity of the damage.

In this section, an efficient damage detection approach is introduced through the combination of the vibration signal processing method and neural network. In order to perform the CEEMDAN-HT-ANN model in damage detection, at first, the vibrations of the steel truss bridge model subjected to a band-limited white noise in the healthy and damaged states are acquired from the identified accelerometer sensors as shown in Figure 3. Afterwards, the vibration responses are decomposed using the CEEMDAN to generate the set of IMFs. Then, the HT is applied to the IMFs to extract their features including instantaneous amplitude (IA), energy, unwrapped phase, and instantaneous frequency (IF). After employing the HHT, a multi-layer perceptron (MLP) neural network is defined to train the relationship between IMFs as the input layers and their four aforementioned features for the healthy state of the bridge as the output layers. The ANN is separately trained based on each feature before applying the damage scenarios on the bridge. The outputs of the CEEMDAN-HT-ANN model based on four aforementioned features extracted from the healthy state of the bridge are compared with those from the scenarios varying in terms of the damage level, and the sensor location relative to the damaged element to classify the severity and detect the location of damage, respectively. Finally, the outputs of the CEEMDAN-HT-ANN model based on IA, energy, unwrapped phase, and IF are evaluated before and after damage, scenarios using different damage indices based on the features captured from the healthy and damaged states of the truss bridge. The defined damage indices indicate the presence, location, and severity of the damage. The framework flowchart of the proposed methodology is shown in Figure 5 and further explanations of this flowchart can be found in Section 4.

## 4. Result and Discussion

### 4.1. Detection of the Presence and Severity of Damage

**Figure 6:**
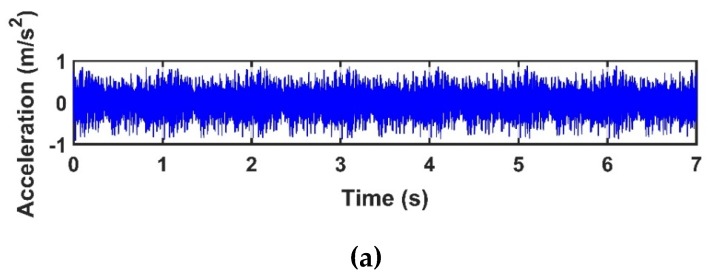

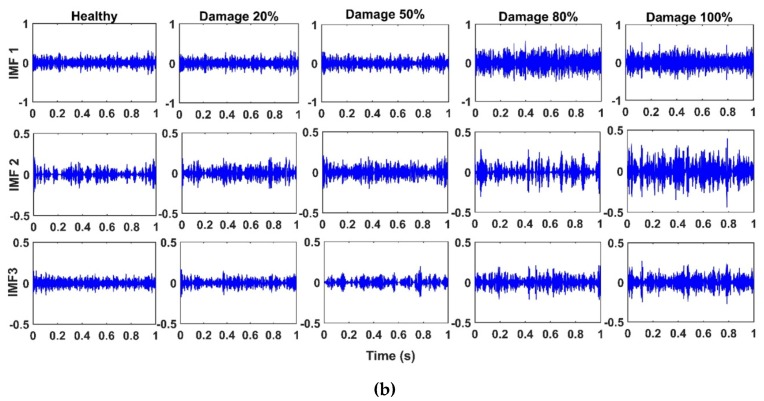
(**a**) acceleration response of sensor-10 for a healthy state of the bridge; (**b**) the first three intrinsic mode functions (IMFs) extracted by the CEEMDAN for sensor-10 for the healthy state and different damage levels.

In this section, the presence and severity of the damage are studied by investigating the results from different damaged states (damage scenarios) of the truss bridge. To this end, sensor-10 is selected as an example checkpoint located nearby the damaged element. Figure 6a,b show a segment of acceleration response of sensor-10 with a duration of 7.0 s before damage (i.e., healthy state), and the first three IMFs extracted by the CEEMDAN for the structure before (healthy) and after damages of 20%, 50%, 80%, and 100%, respectively. The damage of 100% means the removal of the diagonal element from the damage location identified by the dashed line as shown in Figure 3. The first three natural frequencies of the bridge are 19.53, 40.16, and 60.55 Hz, and the corresponding natural period times *T_1_*, *T_2_*, and *T_3_* are 0.051, 0.025, and 0.016 s, respectively. Hence, considering 1.0 s of the acceleration response of the bridge (about 20*T*_1_) is a reasonable time window to analyze in this study.

In Figure 6, the damage spikes are observed in the time-history behavior of the IMFs for the damaged cases of the structure as to be expected. By comparing the first three IMFs, it is observed that the intensity of the spikes of IMFs becomes more pronounced as the level of damage increases. Furthermore, the root-mean-square (RMS) values which represent the total behavior of the IMFs increase by enhancing the damage level. Once the signal is decomposed by the CEEMDAN, the Hilbert Transform (HT) is applied to the IMFs captured from the sensors before and after damage to extract four key features including the energy, IA, unwrapped phase, and IF. Then, the obtained IMFs and the features are used to train the ANN.

**Figure 7:**
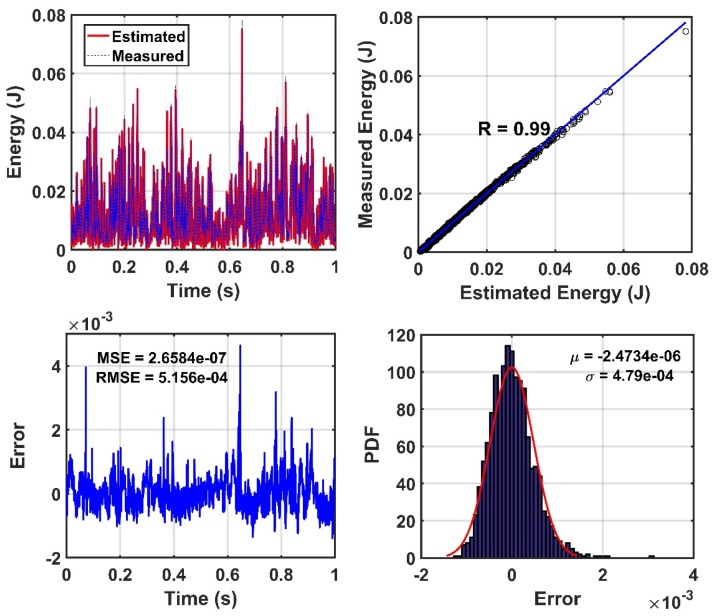
Evaluation of the accuracy performance of training the Artificial Neural Network (ANN) based on the energy parameter of sensor-10 for the healthy state of the truss. (where *R*, *µ*, *σ*, MSE, and RMSE denotes the value of regression, mean, standard division, mean square error, and root mean square error, respectively).

**Figure 8:**
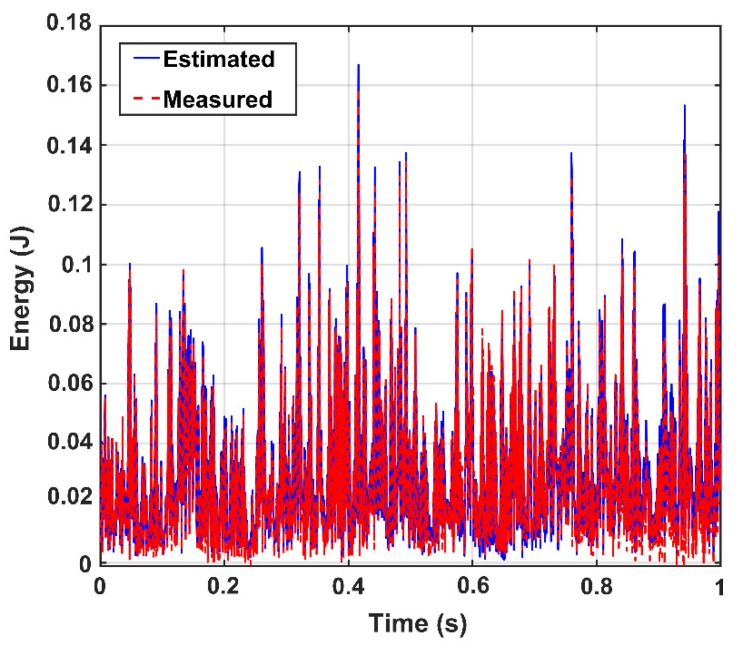
Comparison of the IMFs’ energy of sensor-10 between the outputs of the trained model (i.e., estimated) and the measured data for 100% damage state of the truss.

Before the use of the proposed model, the reliability of the CEEMDAN-HT-ANN model is examined through the correlation and root-mean-error analyses between the measured energy of the IMFs and the estimated (output) energy by the proposed model in the healthy state of the truss bridge. Figure 7 demonstrates the accuracy of the training and testing process of the ANN. In addition, the comparison between the measured energy of the IMFs and the estimated energy by the proposed model for the 100% damage state of the truss is shown in Figure 8. It is found that the CEEMDAN-HT-ANN model can efficiently and accurately perform in estimating the results of both healthy and damaged states of the truss.

**Figure 9:**
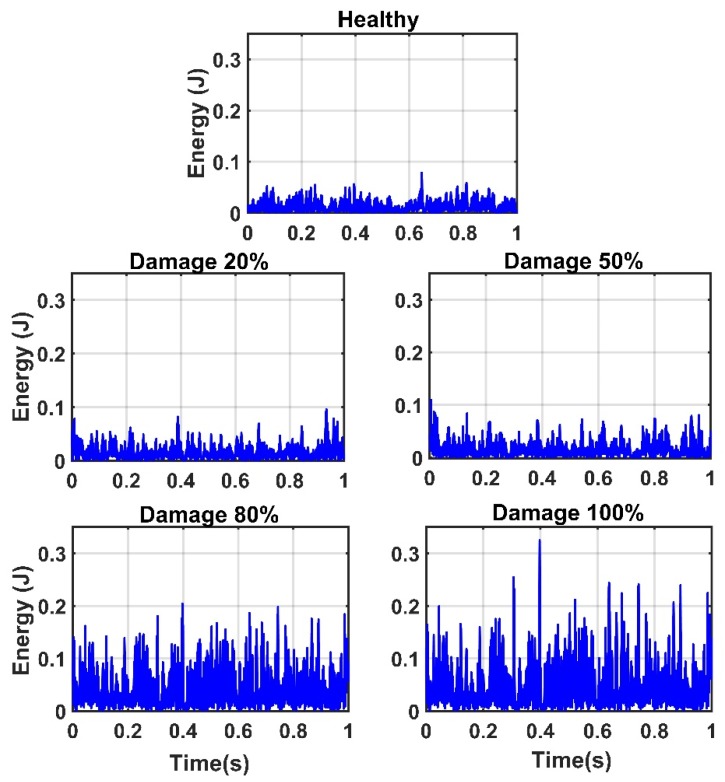
The energy of IMFs of the sensor-10 estimated by the proposed model for the healthy state and different damage levels of the truss.

**Figure 10:**
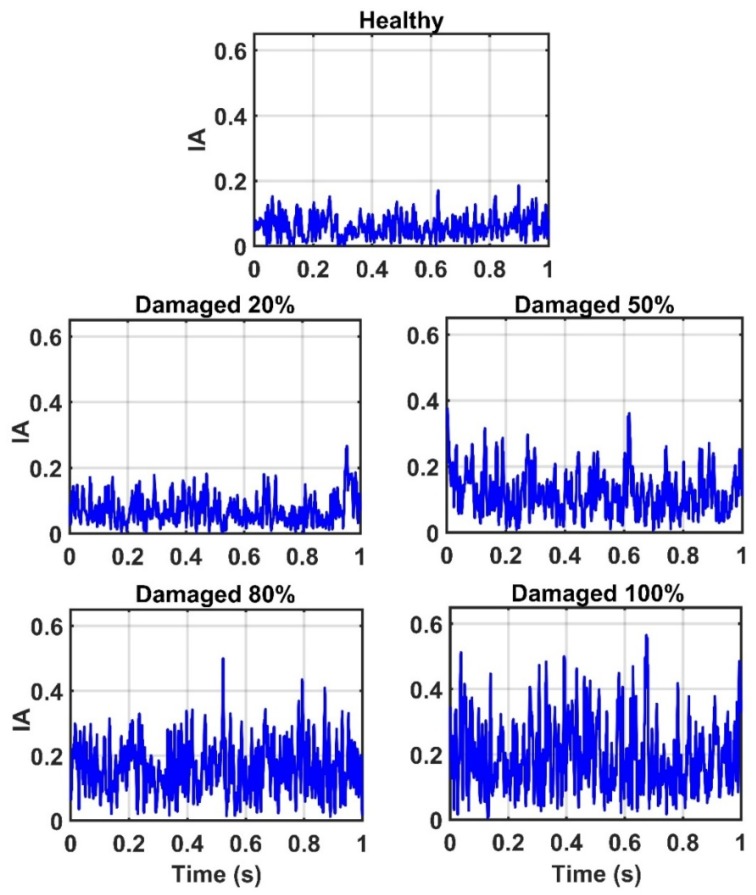
The estimated instantaneous amplitude (IA) of the sensor-10 by the proposed model for the healthy state and different damage levels of the truss.

**Figure 11:**
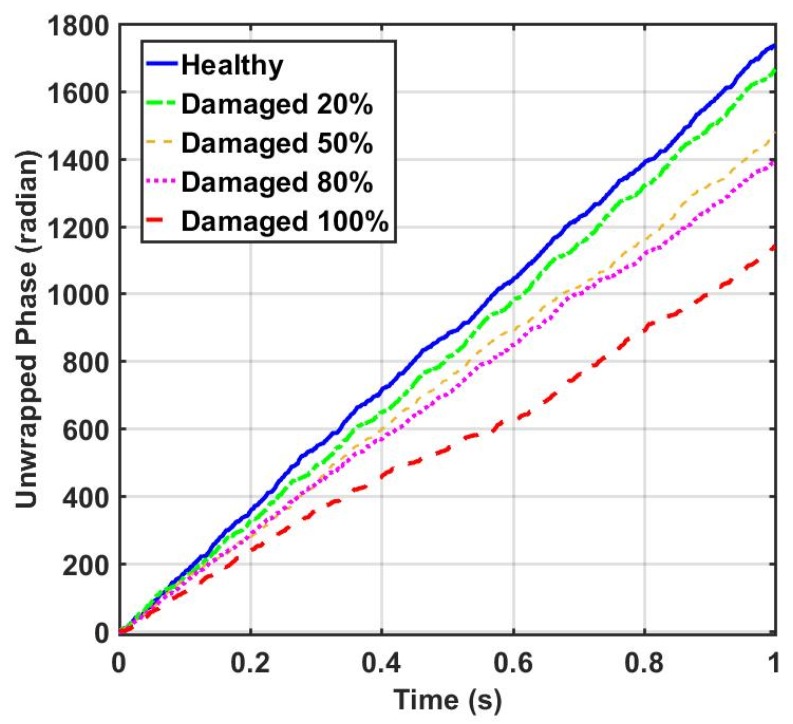
Comparison of the IMFs’ unwrapped phase of acceleration responses of sensor-10 estimated by the proposed model for the healthy and different damage levels the truss.

Due to the successful performance of the ANN, all of the IMFs of thirteen sensors in 20%, 50%, 80%, and 100% damaged states of the structure are used to examine the ANN model. The outputs of the CEEMDAN-HT-ANN model based on the four features (i.e., energy, IA, unwrapped phase, and IF) are compared to show the capability of the proposed model in detecting the presence and severity of the damage in the truss bridge. Figure 9 illustrates the estimated energy using the CEEMDAN-HT-ANN model for the acceleration response of sensor-10 for healthy and different damaged states of the structure. The increase of energy power is observed in proportion to the increase of the damage level. As the second extracted feature, the estimated IA by the proposed model also increases with the increase of the damage level as shown in Figure 10. In addition, the estimated unwrapped phases of the IMFs using the CEEMDAN-HT-ANN model before and after damage states of the truss are depicted in Figure 11. It is found that the unwrapped phase of the IMFs decreases and their deviation increased relative to the healthy unwrapped phase with increasing the level of damage. Consequently, the outputs of the proposed model based on four extracted key features demonstrate the significant positive influences of applying the CEEMDAN and Hilbert transform combined with the ANN in detecting and classifying the damage severity.

The efficiency of the proposed model is more explored by evaluating the results of damage indices based on four features as given in Table 1. The results demonstrate the significant enhancement in the values of the damage indices with increasing the damage level. Furthermore, it is obtained that the damage index values based on the energy feature captured significantly higher values than those from IA and unwrapped phase parameters.

**Figure 12:**
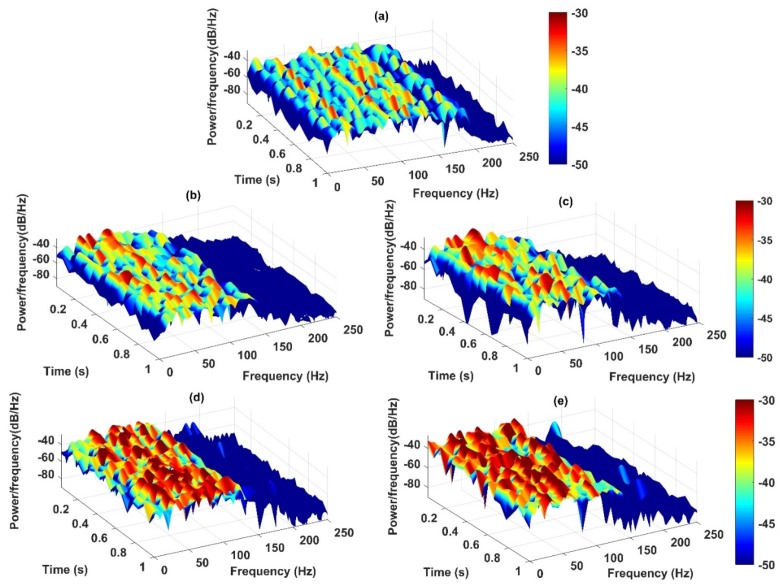
Spectrograms output of the IMFs of sensor-10 under five states of the structure including; (**a**) healthy; (**b**) 20% damage; (**c**) 50% damage; (**d**) 80% damage; and (**e**) 100% damage by the CEEMDAN-HT-ANN model.

The instantaneous frequency is considered as the fourth feature to detect the severity of the damage. Generally, when a structure experiences nonlinear behaviors, changing of structural stiffness causes the change of frequency responses which can consequently imply the existence of damage. The three-dimensional spectrograms of the IMFs are presented in Figure 12a–d. These figures show the high-resolution spectrums of the HHT in which the amplitude of the signal is presented in a time-frequency domain. The sampling frequency of the spectrums is 500 Hz while the length of overlapping windows is 100 samples based on short-time Fourier transform. In Figure 12, it is observed that, when the level of damage increases, the intensity (power) of IF increases in the low-frequency range, especially for 80% and 100% damage levels compared to the healthy state.

### 4.2. Detection of Damage Location

**Figure 13:**
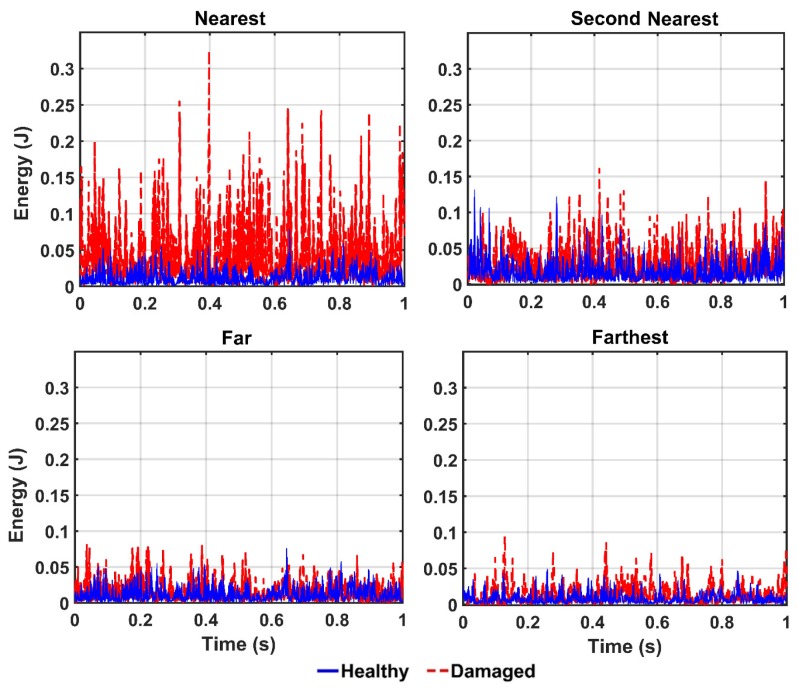
The energy output of the IMFs for different sensor locations from the damaged element by the proposed model.

**Figure 14:**
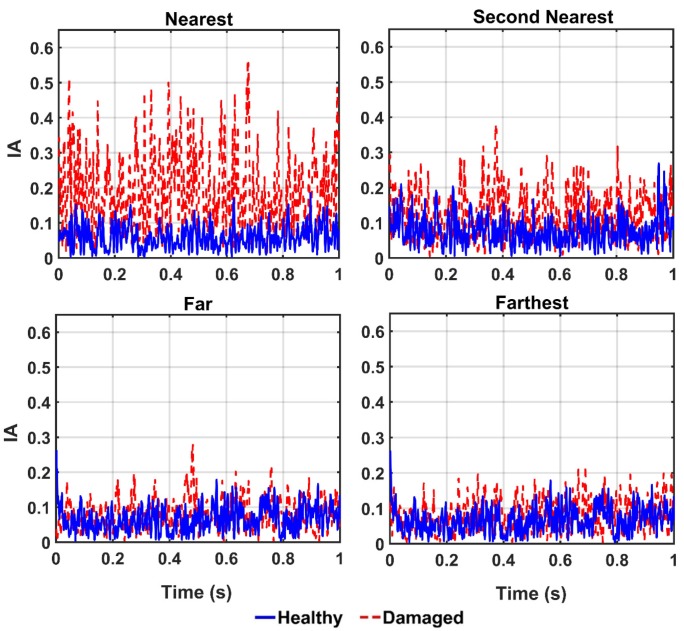
The IA output of the IMFs for different locations from the damaged element by the proposed model.

**Figure 15:**
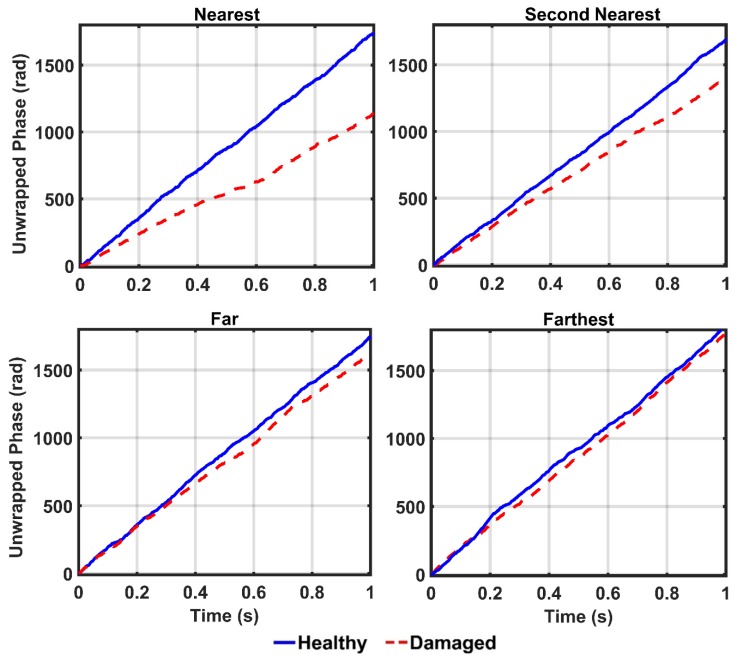
Comparison of the IMFs’ unwrapped phase outputs of the acceleration responses by the proposed model for different sensor locations from the damaged element.

In order to detect the location of damage, the vibration responses of the structure in the healthy and damaged states are captured from four different sensors 10, 9, 4, and 1, which represent the nearest, the second nearest, the farthest, and the farthest sensors from the location of the damaged element, respectively. In this section, the damage scenario is implemented by removing the diagonal element identified in Figure 3 (i.e., damage 100%). Thereafter, the collected vibrations from the aforementioned sensors are decomposed by the CEEMDAN and the four features including energy, IA, unwrapped phase, and IF are extracted from the IMFs. Then, the outputs of the CEEMDAN-HT-ANN model based on these features are quantified to assess the performance of the proposed model in detecting the location of the damage in the truss bridge. Figure 13, Figure 14 and Figure 15 illustrate the comparison of energy, IA, and unwrapped outputs from the proposed model between those obtained for the healthy and damaged states of the truss at different sensor locations relative to the damaged element. It is observed that the intensities of the energy and IA spikes obtained from the sensors located at close distances from the damaged element are greater than those located at far distances. That is, the estimated energy and IA as the outputs of the CEEMDAN-HT-ANN model increase with decreasing the distance of the sensor location from the damaged element that demonstrate the ability of these parameters in detecting the location of the damage. 

In addition, the estimated unwrapped phase as the output of the CEEMDAN-HT-ANN model is assessed to detect the damage location. In Figure 15, it is obviously observed that the value of the unwrapped phase decreases by reducing the distance between the specified sensors and the damaged element and their deviations increase relative to the healthy unwrapped phase. In addition, the damage indices based on the features are given in Table 2. It is found that, although all features are successfully able to locate the damage, the energy-based damage index captures a more efficient approach compared to those based on the IA and unwrapped phase parameters.

**Figure 16:**
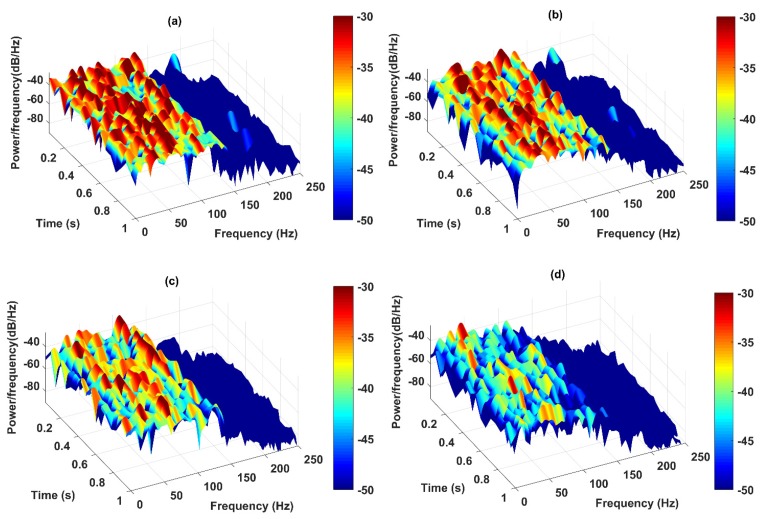
Spectrograms output of the IMFs for different locations of (**a**) the nearest, (**b**) second-nearest, (**c**) far, and (**d**) the farthest sensors from the 100% damaged element through the proposed model.

The instantaneous frequency is considered as the fourth feature to detect the location of damage through the three-dimensional spectrograms. This approach presents the instantaneous frequency of IMFs in a time-frequency domain. The power of IF increases with decreasing the distance of sensors from the damaged element as shown in Figure 16a–d. Upon careful observation of these figures, the spectrogram of the nearest and second-nearest sensors shows the increase of power density in the low-frequency spectra compared to those from far and farthest sensors. That is, there is a decrease in the power of the frequency as the sensor location is moved farther with respect to the damage location. Generally, the extracted features evaluated in this section were successfully able to locate the damage and demonstrate the performance of the proposed methodology both quantitatively and qualitatively in classifying the damage severity and locating the damage.

## 5. Summary and Conclusions

In this paper, the performance of a combined CEEMDAN-Hilbert Transform-Artificial Neural Network (CEEMDAN-HT-ANN) model as a fusion of data analysis and machine learning approach was experimentally assessed in identifying the presence, location, and severity of the damage on a laboratory-model steel truss bridge. To this end, the CEEMDAN was used to decompose the response of the bridge subjected to a white noise excitation. Then, the HT technique was applied to the IMFs before and after the damage to extract the key signal parameters including the energy, instantaneous amplitude (IA), unwrapped phase, and instantaneous frequency (IF). After employing HHT, the IMFs and extracted parameters were selected as the input and output layers of the ANN for the healthy state of the bridge, respectively. Then, the proposed CEEMDAN-HT-ANN model was tested by the IMFs of the damaged truss bridge. The estimated responses of the proposed model based on four parameters were qualitatively compared for different damage scenarios. Moreover, three damage indices were identified based on the estimated output of the proposed CEEMDAN-HT-ANN model under four damage severities and locations as the effective indicators to quantitatively classify the level and detect the location of the damage. By analyzing the experimental results, it was concluded that the damage features of the signals were accurately distinguished and extracted with the proposed approach. In addition, both quantitative and qualitative results showed the capability and robustness of the CEEMDAN-HT-ANN model in addressing the damage location, classifying the severity, and detecting the damage.

## Figures and Tables

**Table 1:** Damage index (DI) results based on the energy, instantaneous amplitude (IA), and unwrapped phase of IMFs for the different damage levels.

IMF Feature	Damage 20%	Damage 50%	Damage 80%	Damage 100%
	DI (%)	DI (%)	DI (%)	DI (%)
**Energy**	23.43	32.72	86.47	94.24
**IA**	21.46	25.88	57.39	62.04
**Unwrapped phase**	6.52	17.13	20.11	41.80

* Intrinsic mode function (IMF), Damage index (DI), Instantaneous amplitude (IA).

**Table 2:** Damage index results based on the IMF features for different sensor locations from the damaged element.

IMF Feature	Nearest	Second Nearest	Far	Farthest
	DI (%)	DI (%)	DI (%)	DI (%)
**Energy**	94.24	35.84	24.92	17.34
**IA**	62.04	51.36	31.35	16.45
**Unwrapped Phase**	41.80	18.90	07.55	05.10

* Intrinsic mode function (IMF), Damage index (DI), Instantaneous amplitude (IA).
